# Psychopathological and clinical-typological aspects of youth chronic endogenous depression

**DOI:** 10.1192/j.eurpsy.2022.940

**Published:** 2022-09-01

**Authors:** V. Kaleda, V. Migalina

**Affiliations:** FSBSI «Mental Health Research Centre», Department Of Youth Psychiatry, Moscow, Russian Federation

**Keywords:** chronic depression, youth, persistent depressive, dysthymia

## Abstract

**Introduction:**

Youth ontogenesis contributes significantly to depressive disorders, causing pronounced atypia, a high level of comorbid pathology. A long-term depressive state lead can to persistent, adverse consequences.

**Objectives:**

To study the clinical, psychopathological and psychometric features of youth chronic endogenous depression (UCED).

**Methods:**

62 patients of the age 16-25 were examined clinically and psychopathologically; the patients were first hospitalized from 2017 to 2020 for a chronic depressive state with non-psychotic mental disorders (ICD-10: F31, F32, F33, F34, F21 keys) lasting more than two years. Psychometric assessment was done by HDRS, SOPS, and SANS.

**Results:**

UCED are characterized by a pronounced atypia with a predominance of symptoms for negative affectivity with apathy, anhedonia, physical and mental asthenia, depressive devitalization. In contrast with non-chronic youth depressions, cognitive disorders, motor inhibition, a large proportion of comorbid pathology are presented in the chronic ones. Depending on the prevalence of additional psychopathological disorders, 2 types were distinguished: Type I – depression with a clear-cut affective psychopathological structure (54.8%, 34 patients); Type II - depression with the symptoms of other than affective registers (45.2%, 28 patients). Psychometric assessment on the HDRS scale, in the sub-scale “negative symptoms” of the SOPS scale, in the sub-scale “anhedonia-associality” of the SANS scale showed a greater severity of psychopathological symptoms in type II depression (p<0.05).

**Conclusions:**

The obtained data confirm the differences between UCED and non-chronic youth depressions and demonstrate the aggravating effect of symptoms of the non-affective spectrum on the severity of UCED and the level of negative affectivity.

**Disclosure:**

No significant relationships.

